# Characteristics of cattle in chronic pens at 17 North American feedlots

**DOI:** 10.3389/fvets.2026.1947975

**Published:** 2026-10-12

**Authors:** Emiline R. Sundman, Renée D. Dewell, Suzanne T. Millman, Karen Schwartzkopf-Genswein, Sarah E. Erickson, Anna K. Johnson, Calvin W. Booker, Tye Perrett, Ryan Rademacher, Daniel U. Thomson, Grant A. Dewell

**Affiliations:** 1 Department of Veterinary Diagnostic and Production Animal Medicine, College of Veterinary Medicine, Iowa State University, Ames, IA, United States; 2 Department of Biomedical Sciences, College of Veterinary Medicine, Iowa State University, Ames, IA, United States; 3 Agriculture and Agri-Food Canada, Lethbridge Research and Development Center, Lethbridge, AB, Canada; 4 TELUS Cattle Professional Services, Okotoks, AB, Canada; 5 Department of Animal Science, College of Agriculture and Life Sciences, Iowa State University, Ames, IA, United States

**Keywords:** animal welfare, compromised cattle, fattening cattle, hospital pen, impaired cattle, reference values

## Abstract

Ill and injured cattle in feedlot chronic pens are an understudied population, and the limited information on this vulnerable population has implications for animal health, welfare, and operational viability. The objective of this study was to describe demographics, diagnoses, and final dispositions of cattle in chronic pens. Seven years of chronic cattle records from 17 United States (High Plains) and Canadian (Alberta) feedlots were retrospectively retrieved from a feedlot veterinary consulting and data-management service. Chronic cattle (“chronics,” *n* = 52,886) were defined as those assigned to a designated chronic pen. Cattle records were evaluated using descriptive statistics. Chronicity rates varied across all feedlots (median 1.1%, range 0.1 to 5%). Across all feedlots, the top three discrete predominant diagnoses were respiratory (median 45.2%, range 19.1 to 99.1%), lameness (median 23.9%, range 0.1 to 50.5%), and bullers (median 2.7%, range 0 to 24.3%). Across all feedlots, the most common final disposition was shipped to slaughter at market weight (median 62%, range 20.1 to 94.1%). Some feedlots shipped (railed) chronics before reaching market weight (median 9.4%, range 0 to 49%). Mortality events consisted of unassisted (median 18.6%, range 5.7 to 37.5%) and euthanasia (median 3.1%, range 0 to 16%) mortalities. Increases in railed and respiratory cattle were seen over the study years, and there was variation in the distribution of respiratory, lameness, and buller Predominant Diagnosis Groups by feedlot arrival season. This is the first large-scale descriptive study of chronic cattle across multiple diagnosis groups, which establishes descriptive reference values that can be used for comparison to better understand chronic pen populations and welfare at similar feedlots.

## 1 Introduction

Promoting and maintaining animal health and welfare are key priorities in beef production systems. For example, the United States (US) National Cattlemen’s Beef Association includes a cattle health and well-being section in their policy book (1), and internationally the World Organization for Animal Health (WOAH) includes a chapter on animal welfare in beef cattle production systems in their Terrestrial Animal Health Code (2). In beef feedlot production, an important population of cattle that may be particularly at risk for negative production and welfare outcomes are chronically impaired cattle (3).

Due to factors such as regional and feedlot-specific variation, there is no standard definition of chronically impaired cattle (“chronic cattle,” “chronics”). The current understanding of chronic cattle is based on a limited number of publications from this research group (3–5) and others (6, 7). Collectively, these studies support the characterization of chronically impaired cattle as those that fail to respond after treatment, have advanced or long-term illness or injury, or need additional support to aid recovery. Chronically impaired cattle are distinct from acutely impaired cattle whose conditions are expected to resolve either through recovery or mortality in a shorter period of time (3). Furthermore, the definition of chronic may vary based on the type of impairment. For example, treatment failure would be defined differently for cattle with non-responsive lameness versus non-responsive Bovine Respiratory Disease (BRD). Both acutely and chronically impaired cattle are often housed in specialized pens, where cattle have easier access to resources such as feed and water and feedlot staff can more frequently monitor and care for them (4, 7). A study from a Saskatchewan feedlot in western Canada reported that 1.3% of calves placed in fall 1998 became chronic (8), suggesting that chronic cattle represented a small percentage the feedlot inventory. In addition to the production and welfare implications related to the prolonged and often non-responsive nature of chronic cattle impairments, proper management of this population can have implications for feedlot operation economic viability, social license to farm, and sustainability (3).

To ensure that animal health and welfare are maintained as well as to identify areas where improvements may be needed, it is imperative to understand the characteristics of populations being managed. Benchmarks are points of reference from which measurements can be made (9). Having benchmarks facilitates continuous quality improvement and can stimulate change within and across organizations (10). For example, one study revealed dairy farmers adjusted calf management practices when provided with benchmarking data from their own farm in relation to aggregate data from other study farms (11). The United States Department of Agriculture (USDA) National Animal Health Monitoring System (NAHMS) 2011 and 2021 beef feedlot studies contained benchmarking data related to hospital pen use and resource provision, but health and management of chronically impaired feedlot cattle were not explored (12).

In the absence of detailed benchmarking data for chronically impaired cattle, the overarching goal of this study was to generate descriptive reference values that can be used to inform and refine chronic cattle care and management. The specific objective of this study was to collect data associated with chronic cattle demographics, diagnoses, and final dispositions across 17 North American feedlots.

## 2 Methods

A large feedlot consulting and data-management service (TELUS Cattle Professional Services, Okotoks, Alberta, Canada) extracted lot- and individual-chronic cattle variables from their consolidated database. This database included data from a chute-side software used by staff at each feedlot to record information on all animal events at the individual animal level. Data pertaining to arrival processing, treatment events, rehandle (revaccination/reimplant) events, and postmortem examination findings are collected according to standardized protocols, allowing for cross-feedlot integration and industry benchmarking. Data from individual feedlots are consolidated daily into a single database across all feedlots.

Since there is not a universally accepted chronic cattle definition, the specific criteria used in this study represent an operational definition intended to facilitate consistent classification. Therefore, chronic cattle were defined as any animal that was housed in a feedlot’s designated chronic pen at some point. While final decisions regarding chronic pen movements are made at the individual feedlot level and may have some variability, the feedlots in this study were managed under the same consulting company which was responsible for training employees on company protocols, including chronic animal management. Under these standardized protocols, considerations for moving cattle to the chronic pen included the number of treatments the animal has received for one condition, the general appearance of the animal, its ability to compete and thrive in a home pen, and whether it requires additional time for convalescence and recovery.

### 2.1 Feedlot inclusion criteria

To be eligible for enrollment, the feedlot’s records had to meet two main inclusion criteria. First, the feedlot placed a consistent number of feeder cattle across all seven study years, with a minimum of 7,000 head per year. This threshold was chosen to ensure sufficient sample size and to maintain geographic representation among participating US feedlots. Second, there were no missing data relating to lot- and individual- chronic cattle variables of interest (Supplementary Table 1). From these eligible yards, a total of 17 feedlots were enrolled in the study and were balanced across the US and CAN. The US (*n* = 8) feedlots were located across four states in the High Plains (Montana, Nebraska, Colorado, and Texas), and the CAN feedlots (*n* = 9) were all located in Alberta, Canada.

### 2.2 Data retrieval and manipulation

Lot- and individual-level records covered a 7-year period (January 1, 2014 – December 31, 2020). Data were extracted from the consolidated database into Microsoft® Office Access (Version 365 ProPlus, Microsoft Corporation, Redmond, Washington, US). For individual animal data, different events such as arrival, treatment, and final disposition were extracted separately to facilitate validation of the individual events before combination into the final dataset. These were cross-referenced against other data sources, such as yard-specific reports. Duplicate values were removed and missing values were queried and added when possible. After these validation steps, all extracted data were combined into one large dataset based on feedlot and animal ID. This dataset was then exported from Microsoft® Office Access to Microsoft® Office Excel (Office 365, Microsoft Corporation, Redmond, Washington, US). Additional variable manipulation in Excel, such as recategorizing existing variables (e.g., creating a treatment diagnosis group variable from the treatment specific diagnosis) and creating new variables from existing variables (e.g., splitting arrival date into separate arrival year and season variables), resulted in a total of 46 variables of interest (Supplementary Table 1).

### 2.3 Data filtering and exclusion criteria

To obtain the final dataset used for the descriptive analysis, data were filtered in three steps (Figure 1). First, lot-level animal records were pulled for all lots during the study years. Second, individual-animal records for chronic cattle from these lots were pulled. Third, individual chronic cattle treated a single time as a buller with no additional treatment events (single event bullers, SEBs) were excluded (*n* = 15,759). Buller cattle are those impacted by buller-steer syndrome (BSS) and defined as an animal that is repeatedly mounted/ridden by other cattle (13). A typical buller treatment is to remove the affected animal from their home pen into a separate specialty pen (see Supplementary Table 1). During initial data visualization, four feedlots were noted to have abnormally high prevalence (10% or more of the chronic cattle population) of SEBs. These buller cattle would typically be considered acutely impaired, as no follow-up treatments were given after the first treatment. Conversely, bullers that received two or more treatments were considered multiple event bullers (MEBs), which indicated the buller condition was not resolved after the first treatment event and may have been a chronic issue. Consultation with TELUS veterinarians indicated a primary reason for discrepancies in SEBs is that some feedlots had a separate buller and chronic pens, while others placed SEBs into their chronic pens. Thus, feedlots without separate buller and chronic pens would have an increased number of SEBs. To minimize the placement discrepancies for likely acutely impaired SEBs into chronic pens across feedlots, these animals were removed from the dataset.

**Figure 1:**
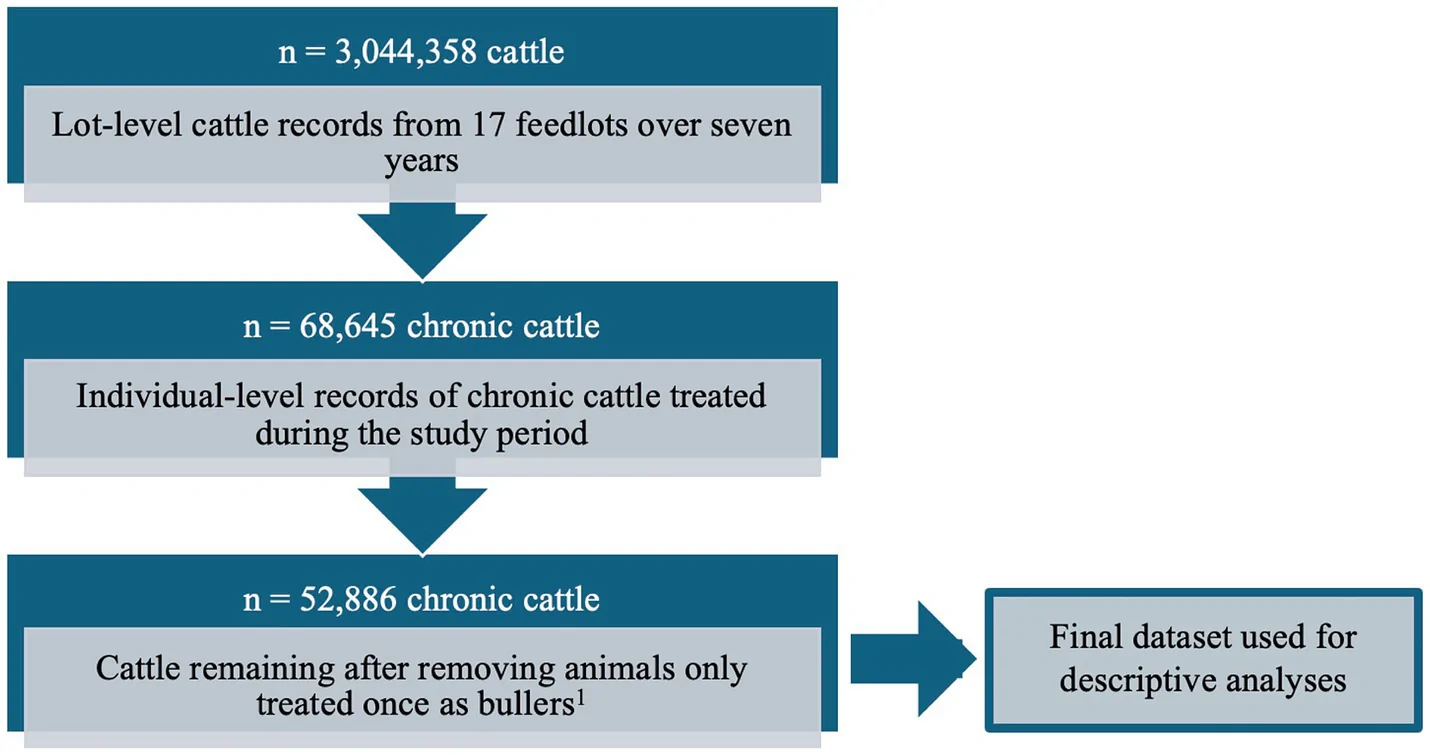
Flow chart of data filtering conducted to obtain the dataset used in this study. ^1^After consulting with TELUS veterinarians, any chronic cattle with a single buller treatment and no other treatment events were removed to minimize skewed outcomes from unusually high one-treatment buller populations.

### 2.4 Primary outcomes

The main descriptive outcomes of interest were predominant diagnosis group (PDG) and final disposition. The PDG outcome consisted of seven categories: respiratory, lame, metabolic, musculoskeletal, buller, other, and multiple (see Supplementary Table 1 for details). Specific diagnoses were assigned at each treatment event by trained feedlot personnel using standardized diagnostic criteria established by the consulting veterinary service, with ongoing oversight from consulting veterinarians. Each specific diagnosis was subsequently classified into one of the diagnosis groups defined in Supplementary Table 1. For each animal, the number of treatment events attributed to each diagnosis group was summed, and the diagnosis group with the greatest frequency was assigned as the animal’s PDG. If two or more diagnosis groups occurred an equal number of times and shared the highest frequency, the animal was classified as “multiple.” Lameness and musculoskeletal conditions were considered separate PDGs. Lameness consisted of disorders affecting the feet, hoof, or joints, whereas musculoskeletal conditions consisted of injuries attributed to facilities, equipment, or other external causes.

The final disposition outcome included three final dispositions: ship, rail, and mortality (see Supplementary Table 1 for details). Rail (sometimes also called “realized”) was defined as shipping cattle for slaughter prior to reaching optimal slaughter weights.

### 2.5 Calculations

Chronicity rates were calculated for each feedlot during the study period as:

$$\frac{\#\text{of chronic cattle}}{\text{total feedlot population}}$$

Treatment rates were calculated for each feedlot during the study period as:

$$\frac{\mathrm{lot}\;\text{level treatment events for}\;\mathrm{all}\;\text{PDGs}}{\text{total feedlot population}}$$

This treatment rate may include counts of individual cattle that received treatments in more than one PDG, and thus are an overestimation compared to feedlot morbidity rates, which would only include individual cattle once, regardless of PDG.

The percentage of cattle fed that were steers at each feedlot was calculated as:

$$\frac{\#\text{of steers}}{\text{total feedlot population}}$$

The percentage of cattle placed that were calves at each feedlot was calculated as:

$$\frac{\#\text{of calves}}{\text{total feedlot population}}$$

Arrival seasons were calculated from arrival dates using astronomical season boundaries, with spring beginning on March 20, summer on June 20/21, fall on September 22/23, and winter on December 20/21 (depending on the year).

### 2.6 Statistical analysis

All descriptive statistics and figures were obtained using Microsoft Excel or R (v4.4.2; R Core Team 2024). Descriptive statistics included primarily percentages, medians, and ranges. Where relevant, means ± standards deviations are also presented. Feedlot-level descriptive demographics are presented for all 17 feedlots. Descriptive results are presented for the entire final dataset.

## 3 Results

The final validated dataset included lot-level data from 11,956 lots representing 3,044,358 head of cattle, and individual-level chronic cattle data representing 52,886 chronics.

### 3.1 Descriptive demographics of chronic cattle from 17 feedlots

#### 3.1.1 Feedlot-level demographics

Descriptive statistics for the 17 feedlots are in Table 1. Feedlots placed 23,909 head/year (range 8,227 to 65,436). The number of chronic cattle contributed by each feedlot ranged from a minimum of 51 chronics in 40 lots, to a maximum of 21,468 chronics in 879 lots. The feedlot-level chronicity rates ranged from 0.1 to 5% (median 1.1%). Feedlot-level treatment rates for all conditions ranged from 8.3 to 30.3% (median of 17.7%). Feedlot-level respiratory treatment rates ranged from 4.4 to 23.0%, (median 10.4%). The average lot arrival weights at each feedlot ranged from 174 kg to 383 kg, (median 303 kg). Overall more calves were placed than yearlings, with a median of 69.1% calves (range 11.8 to 100%) placed across feedlots. All but two feedlots fed more steers than heifers, with a median of 67.3% steers (range 46.1 to 100%). At the lot-level, more beef-breed cattle were placed than dairy or dairy-cross breeds (median 93.9% beef-breed, range 23.8 to 100%).

**Table 1:** Feedlot-level demographic data for the eight United States (US) and nine Canadian (CAN) feedlots from 2014 to 2020.

Feedlot ID	Country^1^	Average head/year	# Lots w/Chronic cattle^2^	# Chronic cattle	Feedlot chronicity rate^3^ (%)	Feedlot treatment rate^4^ (%)	Feedlot mortality rate (%)	Lot arrival weight (avg; kg)	% Steers^5^	% Calves^6^
1	US	36,524	651	6,683	2.6	23.8	9.5	214	60.3	79.3
2	US	13,370	103	2,759	2.9	30.3	6.1	235	81.5	99.3
3	CAN	13,505	75	1,446	1.5	17.0	2.0	321	100	43.1
4	CAN	23,909	336	958	0.6	8.3	2.0	383	60.7	31.1
5	CAN	36,694	374	3,334	1.5	20.8	2.7	337	78.9	69.1
6	CAN	8,227	91	1,734	3.2	19.8	2.9	312	59.8	66.8
7	CAN	15,931	139	753	0.7	28.1	4.8	174	97.9	100
8	CAN	27,539	435	1,580	0.8	10.7	2.2	364	46.1	30.9
9	CAN	34,580	338	2,475	1.0	14.5	3.7	351	61.3	54.0
10	CAN	27,037	208	1,937	1.1	16.9	3.8	320	81.6	69.5
11	CAN	9,792	224	1,299	2.0	14.9	1.8	303	48.6	74.9
12	US	41,538	194	1,669	0.6	21.4	10.2	228	54.3	87.0
13	US	12,842	69	337	0.4	9.7	4.5	294	57.5	65.9
14	US	9,218	40	51	0.1	21.4	1.4	316	67.3	11.8
15	US	61,078	879	21,468	5.0	19.5	6.0	193	79.4	80.9
16	US	65,436	561	3,284	0.7	10.3	3.3	276	80.4	24.5
17	US	10,233	295	1,119	1.6	17.7	3.4	288	83.9	84.1
Avg ± SD (med)	8 US 9 CAN	26,321 ± 17,732 (23,909)	295 ± 232 (224)	3,111 ± 4,971 (1,669)	1.5 ± 1.27 (1.1)	18.0 ± 6.24 (17.74)	4.1 ± 2.55 (3.4)	289 ± 61 (303)	70.6 ± 16.2 (67.3)	63.1 ± 26.4 (69.1)

1 Feedlots in CAN were located in the Alberta region. Feedlots in the US were located in the High Plains region.

2 The number of lots at each feedlot that contained at least one chronic animal that was included in the chronic cattle dataset.

3 Chronicity rate was calculated as the (# of chronic cattle) / (total feedlot population) during the study period for each feedlot.

4 To calculate treatment rate, the total number of treatments in each predominant diagnosis group (PDG) given to individuals at the lot level were summed to represent the total number of treatments for each lot of cattle. Then, treatment rate was calculated as the (lot-level treatment events for all PDGs) / (total feedlot population) from the lot-level data for each feedlot during the study period. These values may include counts of individual cattle that received treatments in more than one PDG, and thus are an overestimation compared to feedlot morbidity rates, which would only include individual cattle once, regardless of PDG.

5 The percent of cattle fed that were steers, calculated as (# of steers) / (total feedlot population) during the study period for each feedlot.

6 The percent of cattle fed that arrived as calves, calculated as (# of calves) / (total feedlot population) during the study period for each feedlot.

#### 3.1.2 Chronic cattle demographics

##### 3.1.2.1 Predominant diagnosis groups

The predominant diagnosis groups for chronic cattle at each feedlot are presented in Figure 2. Across all feedlots, the most common PDG was respiratory disease (median 45.2%, range 19.1 to 99.1%), followed by lameness (23.9%, 0.1 to 50.5%). Feedlots had 8.8% (0.5 to 15.7%) of chronics with the multiple PDG. The remaining PDGs were 5.1% (0 to 18.4%) other diagnoses, 2.7% (0 to 24.3%) bullers, 2.7% (0 to 16.6%) metabolic, and 2% (0 to 25.7%) musculoskeletal PDGs.

**Figure 2:**
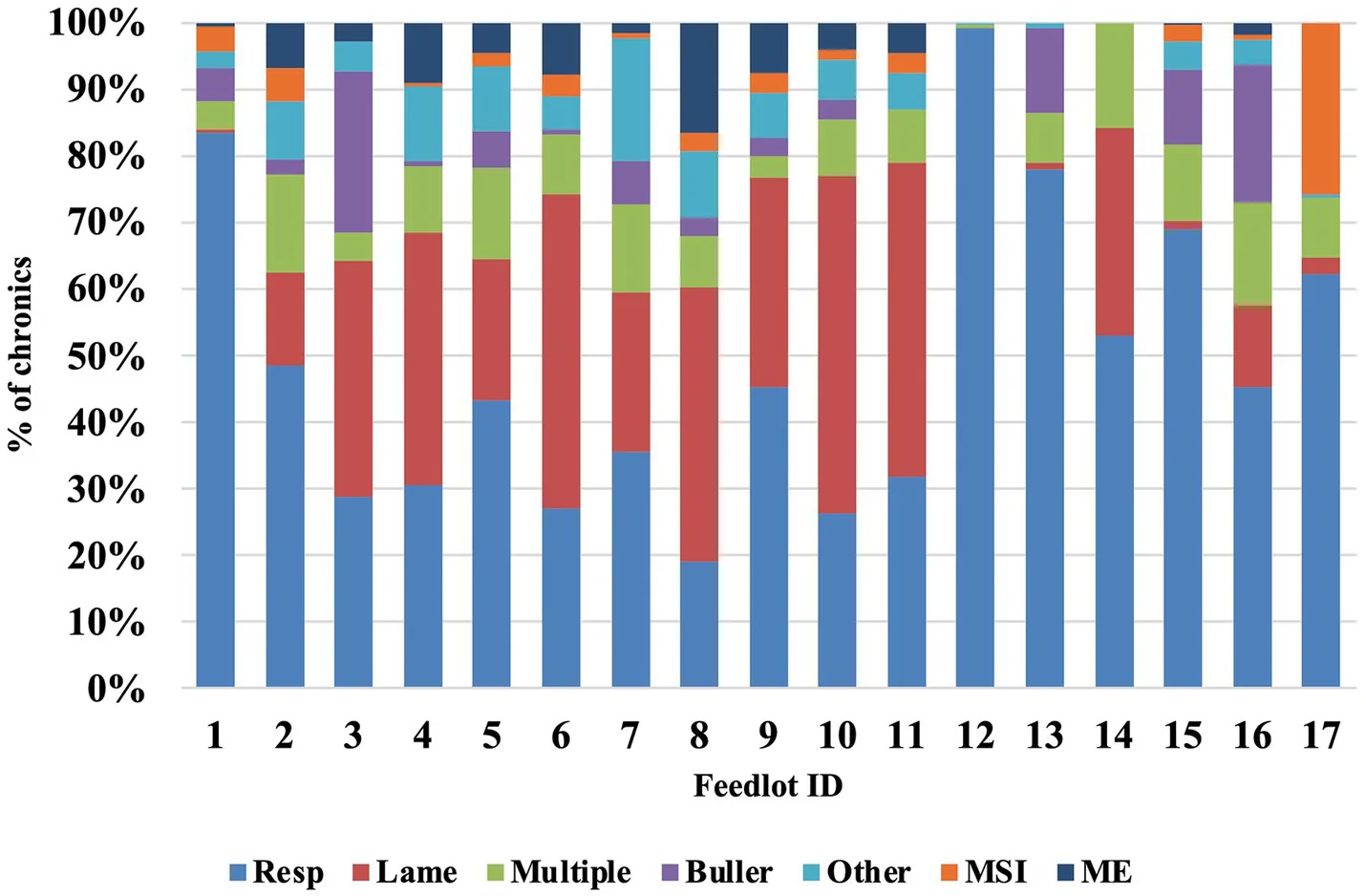
Distribution of chronic cattle predominant diagnosis groups (PDG) across the 8 United States and 9 Canadian feedlots in the dataset. PDGs are respiratory disease (Resp), lameness (Lame), treated equal times for multiple conditions (Multiple), persistently ridden animal (Buller), treatments not covered under the other categories (Other), musculoskeletal conditions (MSI), and metabolic conditions (ME). For specific diagnoses under these categories, see methods.

##### 3.1.2.2 Final dispositions

The final dispositions for chronic cattle at each feedlot are presented in Figure 3. Feedlots shipped 62% of chronic cattle at market weight (range 20.1 to 94.1%) with an average of 418 days on feed (DOF; days from feedlot placement to final disposition). Feedlots railed 9.4% of chronic cattle, but this value varied greatly between feedlots (0 to 49%, interquartile range 6 to 14.9%). Railed chronic cattle spent an average of 202 DOF before railing. When evaluating by region, 6/9 (67%) Alberta feedyards had rail rates above the median, and 6/8 (75%) High Plains feedyards had rail rates at or below the median. Within chronic cattle that were railed, 37.2% had a respiratory disease PDG, 24.8% were lame, 11% were treated for multiple conditions, 7.9% for other conditions, 7.6% for musculoskeletal conditions, 6.4% as bullers, and 5.1% for metabolic conditions.

**Figure 3:**
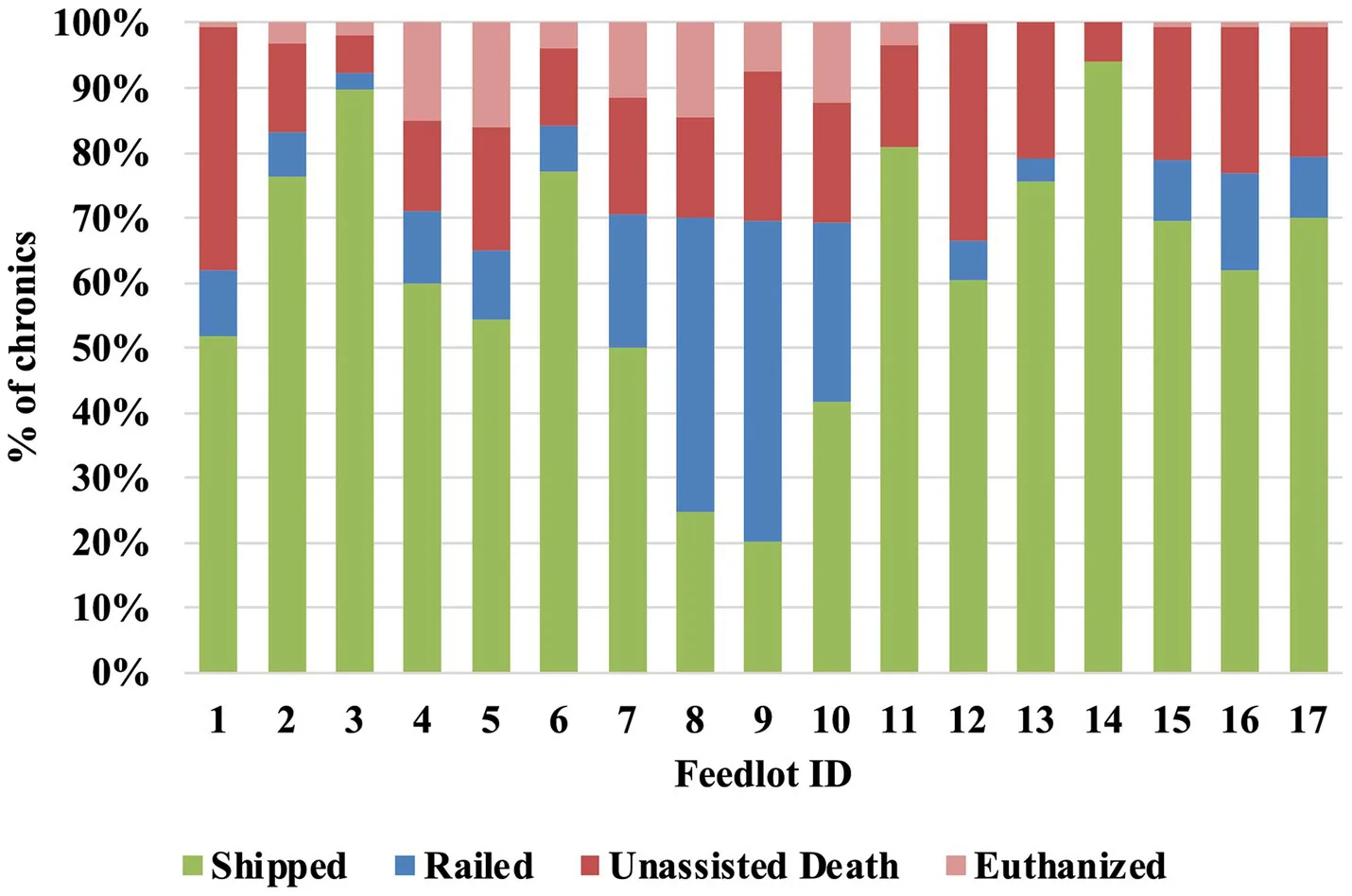
Distribution of chronic cattle final dispositions across the 8 United States and 9 Canadian feedlots in the dataset. Final dispositions are shipped (sent to market at full market weight), railed (sent to market before reaching full optimal market weight), euthanasia mortality (assisted), and unassisted mortality.

Across feedlots, the median proportion of chronics with a PM (post-mortem; mortality) final disposition was 23.1% (5.9 to 38.2%). These PM cattle spent an average of 134 DOF before death. Chronic mortalities can alternatively be presented as 18.6% (5.7 to 37.5%) of chronics with unassisted mortalities (UM), and 3.1% (0 to 16%) with euthanasia mortalities (EM). Considering only the PM chronics, this breaks down into 18.6% (0 to 52%) of PMs resulting from euthanasia, and the remaining 81.4% (48 to 100%) from UDs.

##### 3.1.2.3 Patterns in PDG and final dispositions by year and season

The distribution of the chronic cattle predominant diagnoses and final dispositions by arrival year and arrival season can be found in Figure 4. The number of chronic cattle identified across all study years ranged from 5,796 to 9,800 per year, with an average of 7,555 ± 1,308 chronics each year. Across seasons, the greatest number of chronics were placed in the fall, followed by spring, then winter, and the fewest in the summer (17,309, 13,108, 12,002, and 10,467 chronics placed by each season, respectively).

**Figure 4:**
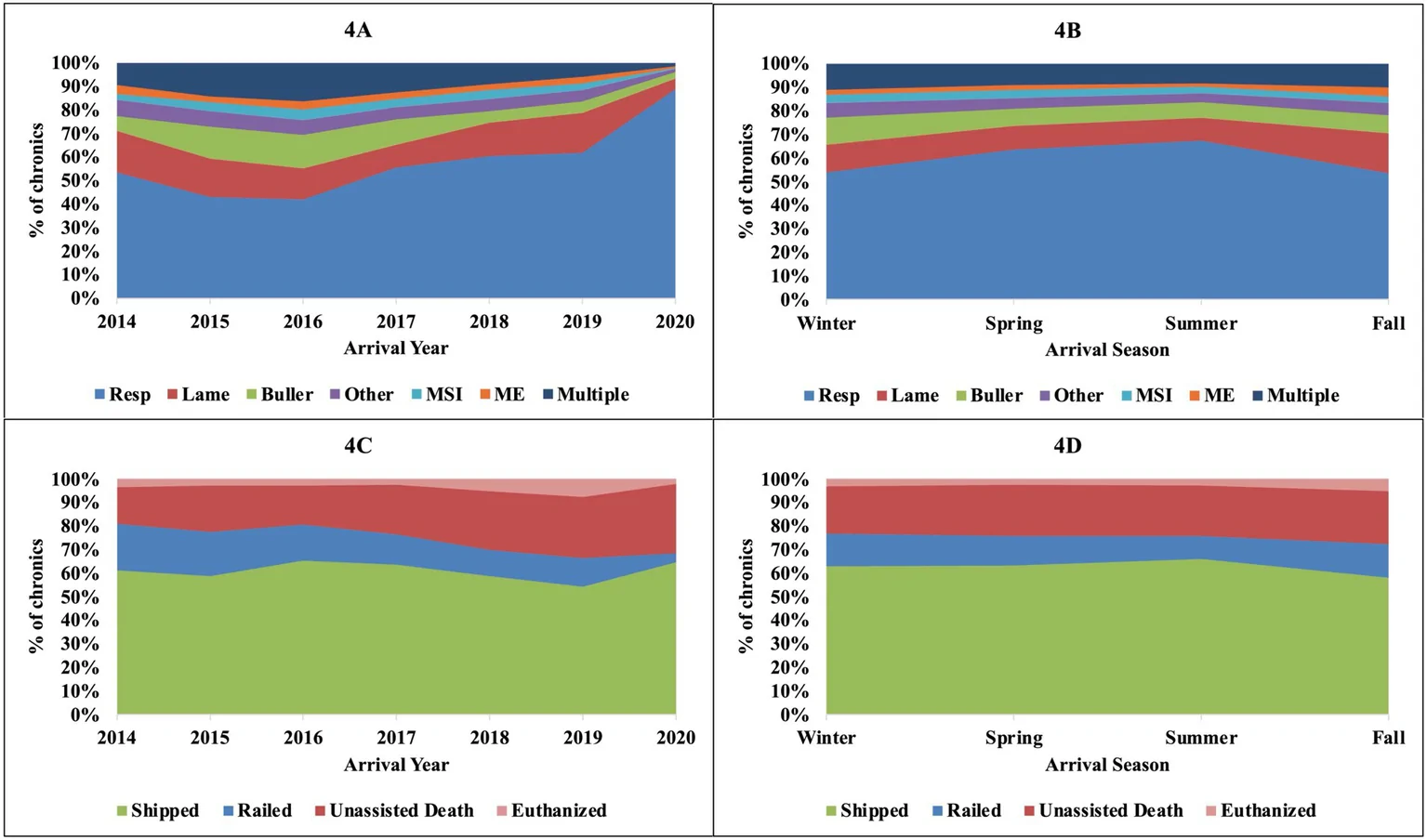
Predominant diagnoses groups and final dispositions of chronics by arrival year and arrival season. PDGs **(A,B)** are respiratory disease (Resp), lameness (Lame), treated as a persistently ridden animal (Buller), treatments not covered under the other categories (Other), musculoskeletal conditions (MSI), metabolic conditions (ME), and treated equal times for multiple conditions (Multiple). For specific diagnoses under these categories, see methods. Final dispositions **(C,D)** are shipped (sent to market at full market weight), railed (sent to market before reaching full optimal market weight), euthanasia mortality (assisted), and unassisted mortality.

## 4 Discussion

This study presents data specific to 52,886 chronic cattle across 17 North American commercial feedlots. Overall, chronicity rates varied across feedlots in this study. Similarly, there was feedlot-level variation in PDG, with respiratory disease and lameness as the two most common conditions. Shipment to slaughter at market weight was the most common final disposition, although ship versus rail rates were variable at individual feedlots. Mortality levels were relatively consistent, with unassisted mortalities more common than euthanasia mortalities. Taken together, while most chronic cattle ultimately reached market weight, railing and mortality were important outcomes with implications for both production efficiency and animal welfare. Finally, increases in railed and respiratory cattle were seen over the study years, and there was variation in the distribution of respiratory, lameness, and buller PDGs by feedlot arrival season. Collectively, these findings provide descriptive reference values that may serve as preliminary benchmarks for chronic cattle populations managed in comparable commercial feedlots and contribute important context to better understand production outcomes and welfare considerations for cattle managed within chronic pens.

### 4.1 Chronicity and treatment rates

Overall, calculated chronicity rates in this study (median 1.1%) are comparable to the 1.3% chronicity rate reported for fall-placed calves in a 1998 study at a single large feedlot in central Saskatchewan (8). Since lightweight cattle are at higher risk for respiratory disease (7, 14), the most common chronic PDG in this study, feedlots with lower arrival weights could be hypothesized to have higher chronicity rates. However, while the two feedlots with the highest arrival weights had below average chronicity rates, the two feedlots with the lowest arrival weights had both substantially above and below average chronicity rates. Thus, descriptive evidence for this hypothesis was mixed and warrants further investigation.

The 17.7% median treatment rate at feedlots in this study is likely an overestimation of traditional morbidity rate, since individual cattle that received treatments in more than one PDG would be included multiple times in the calculation. In a study evaluating relationships between feed delivery data and total feeding period morbidity across 10 US feedyards, overall cohort-level morbidity was classified into high (≥15%) and low (<15%) categories (15). Although treatment versus morbidity rates are not directly comparable, the threshold provides useful context for interpreting the magnitude of treatment rates observed in this study. For respiratory treatment rate, the median value (10.4%) in this study is lower than that reported in the USDA NAHMS 2011 data, where 16.2% of cattle placed were affected by respiratory disease (16). For cohort-level general and respiratory treatment rates, as well treatment events in individual chronic cattle, an important consideration is antimicrobial stewardship. An Australian feedlot cattle study found that injectable antimicrobials are most frequently used to treat respiratory disease (17), the most common PDG in this study. While antimicrobial use data was not available in this study, repeated treatment events likely relate to repeated antimicrobial exposures for some cattle. Incorporating antimicrobial use data in future chronic cattle studies could help evaluate possible relationships between treatment events, chronicity, and antimicrobial stewardship.

### 4.2 Predominant diagnosis groups

Respiratory disease was the most common PDG for chronic cattle in this study, which is consistent with respiratory disease being the primary cause of morbidity in US (16) and CAN (18) feedlots. The respiratory PDG prevalence is also within the range of previous reports of respiratory disease in chronic pens, including 49% at a single large Saskatchewan feedlot (8), 35.7% at three southern Alberta feedlots (19) and 57% at five Iowa feedlots (20). Lameness was the second highest PDG in our study at 23.9%, which is lower than the lameness prevalence reported as 48.5% (8), 37.4% (19) and 33% (20) of chronic cattle diagnoses in the other studies. By region, there was a descriptive difference in the most prevalent PDG. In just the Alberta feedlots, lameness accounted for 36.2% of chronic cattle, which is closer to the previously reported values. Conversely, three of the High Plains feedlots did not even have lameness in their top three PDGs. It is possible that differing pen conditions, climate, and weather conditions between regions could have influenced chronic lameness rates. In a study of southern Alberta feedlots, lameness was found to be greatest in the spring, and odds of becoming lame were increased with increased precipitation and temperature range (21). Another study found that weather conditions are likely related with increased mud depth, which was considered lameness risk factor (5). However, these factors were not available for the data in this study and therefore cannot be evaluated in relationship to the lameness PDG.

Despite the exclusion of SEBs, considerable variation in buller PDGs remained across feedlots. Management factors that could not be evaluated in this study are a likely source of some of this variation. Buller-steer syndrome is a unique behavioral issue, and typical treatment involves removing the animal to separate pen for a few days of rest and recovery before being returned to the home pen. If relapse occurs, the buller may be moved long-term to a buller pen (22, 23). Some feedlots in this study had separate chronic and buller pens, while others did not. Additionally, cattle with BSS may be at higher risk of treatment for secondary conditions such as respiratory disease (22), which could complicate record keeping for cattle with concordant conditions. Thus, difference in buller management and record-keeping may have influenced the study population and contributed to the buller PDG variation among feedlots. Biologically, feedlot heifers are typically fed melengestrol acetate (MGA), which prevents ovulation and resultant estrus behaviors such as mounting (24). Only seven of the 4,310 bullers in this study were heifers, and since feedlots varied in the number of heifers vs. steers fed, this may also help explain the variation in bullers across feedlots.

### 4.3 Final dispositions

Most cattle (62%) that entered the chronic pens in this study recovered and were eventually shipped at market weight, which is the ideal outcome for the feedlot and cattle welfare. Chronic cattle could have either shipped directly from the chronic pen or could have been returned to a home pen to finish out. Similarly, other studies report that most chronic cattle are eventually shipped at market weight. Pollock and colleagues found that 60% of cattle that entered a chronic pen at a large Saskatchewan feedlot were returned to a home pen (8). In a study of Iowa feedlots, 83% of cattle were sent to slaughter from the chronic pen or returned to a home pen (25).

Railed animals are sent to slaughter before reaching full market weight, typically due to chronic conditions, injury, or overall poor performance (6, 26). Rail rates varied considerably among feedlots in this study and were descriptively higher in Alberta versus High Plains feedlots. Regional differences in proximity to facilities that receive railers could impact rail rates. Economically, railing cattle is typically associated with a net loss on investment (6). Importantly, our data indicated that shipped chronic cattle spent on average over 200 more DOF than railed chronic cattle. It is possible that when substantial time, resources, or other inputs needed to reach full market weight, railing may more attractive. Animal diagnosis can also impact railing decisions. Across all feedlots the most common PDG in railer cattle was respiratory disease, followed by lameness, which is consistent with previous studies reporting reasons for railing feedlot cattle (26–28). However, the broad classification of PDGs in this study limits an in-depth evaluation of how diagnosis impacts railing decisions. Finally, the transport of unfit (sick, emaciated, debilitated) cattle is listed as one of the most significant welfare concerns during transport (29). Animals that are weak, diseased, or injured may already be experiencing welfare issues such as pain and sickness and may be less able to cope with transport challenges (30). In both the US and Canada, regulations and industry guidelines outline restrictions on the transport of unfit or compromised cattle, including cattle with severe lameness or emaciation (31–33). Although railer cattle should be fit for transport at loading, they may be at risk for becoming non-ambulatory during the transport process and thus may be condemned at plant arrival. Research is needed to help clarify these outcomes to inform decisions about railing cattle.

Mortality rates were the most stable disposition from chronic pens in this study. The median mortality rate (23.1%) fell within previously reported values, which vary from 17% (25) to 40% (8). When splitting into unassisted versus euthanasia mortalities, there was considerable feedlot-level variation, and more cattle experienced UM than EM. This distinction has implications for animal welfare. While unexpected mortalities can occur in chronic pens, cattle in these pens are closely monitored and thus euthanasia rates are expected to be higher. However, euthanasia decisions in chronic cattle with slowly progressing conditions can be difficult. These findings highlight the importance of detailed written veterinary protocols and training related to timely euthanasia, and indicate that further research and extension efforts in this are still needed. In US and CAN feedlot audits, failing to follow veterinary protocols related to timely euthanasia of critically ill, distressed, or injured animals is considered an egregious act of neglect that can result in feedlot audit failure (34, 35).

### 4.4 Fluctuations by year and season

Descriptively, there was a five-fold decrease in proportion of chronic cattle railed over the seven study years. Interestingly, there was also a 1.7-fold increase in the proportion of cattle with respiratory PDG during this period. While the present study did not evaluate whether these observations were related, previous surveys of feedlot veterinarians similarly identified chronic BRD a common condition among railer cattle, with the percentage of respondents identifying chronic BRD as the primary railer diagnosis increasing from 43% in 2011 to 73% in 2015 (27, 28). Factors underlying this and other descriptive patterns in this dataset, such as employee turnover, management changes, outbreaks of certain diseases, unusual weather patterns, etc., could not be evaluated in this study. Cattle prices may also impact decisions to ship, rail, or euthanize chronic cattle. Notably, cattle prices remained relatively stable throughout most of the study period and began increasing substantially only in 2021 (36). Therefore, the yearly disposition outcomes in this study may not fully reflect decision-making under more recent market conditions.

The distribution of respiratory, lameness, and buller PDGs varied descriptively by feedlot arrival season. While respiratory PDG was consistently high in all seasons, there was a 14% higher proportion of respiratory chronics in cattle that arrived at the feedlot in the summer versus winter or fall. This is partly consistent with previous feedlot studies reporting higher odds of developing BRD among Australian cattle placed in the summer and fall versus spring (37), and greater BRD morbidity incidence rate ratios in US cattle placed in the summer after traveling long distances versus winter placed cattle (38). For lameness PDG, the proportion of lame chronics was descriptively 1.75-fold higher in fall versus summer placed cattle. This is inconsistent with a previous lame cattle study at 28 Alberta feedlots that reported the lowest overall lameness in fall placed calves, and highest in yearlings (39). However, different lameness types (i.e., footrot, injury, joint infection) can have different seasonal distributions (5, 39). Since lameness was defined broadly in this study, specific lameness conditions and their seasonal prevalences could not be evaluated. The buller PDG was highest in winter placed cattle in this study, although buller events occurred throughout all seasons. This agrees with previous work suggesting that buller-steer syndrome can flare up at any time in the feeding period, regardless of season, and may be impacted by stressors such as hot weather or commingling events (13, 40). Interpretation of PDG and feedlot arrival season should also consider possible delays between feedlot arrival, disease development and treatment, and movement to a chronic pen. Additionally, diagnosis-specific timing of disease development and treatment could play a role; BRD typically occurs earlier in the feeding period (41), whereas lameness timing varies with different lameness types (42).

### 4.5 Study limitations

One limitation of this study was our definition of chronic cattle according to their placement in a chronic pen at some point during the feeding period, which differs from some other uses of the term. Second, results from this study reflect the specific characteristics of the enrolled feedlots which were advised by the same veterinary consulting company. An advantage of this is greater consistency in management, training, and protocols related to important outcomes such as treatment and diagnosis. Furthermore, variation among individual feedlots is still evident in the study data. A primary limitation is that these feedlots represent a small sample of the highly diverse feedlots in North America, and caution should be exercised for direct extrapolation to feedlots with different management systems or characteristics. These results are best interpreted descriptive reference values that can be used to inform and refine chronic cattle care and management. Additional data from other types of feedlots would be helpful to further develop benchmarks. Third, some granularity was lost by grouping data into general categories for comparison purposes. For example, our seven PDG variables generalized over 30 unique diagnoses that may have different etiologies and/or impacts on chronic cattle outcomes and welfare. Finally, the retrospective nature of this study disallowed the evaluation of certain unavailable factors such unusual events, employee changes, climate/weather, and pen conditions on chronic cattle populations. These are important factors that should be investigated in future studies.

## Data Availability

The datasets presented in this article are not readily available because they were obtained from a third-party company and are not publicly available due to proprietary restrictions. Requests to access the datasets should be directed to TELUS Cattle Professional Services - Okotoks.

## Author’s note

Portions of this manuscript have previously appeared as part of E. R. Sundman’s doctoral dissertation submitted in partial fulfillment of the requirements for the Population Sciences in Animal Health degree at Iowa State University (43).
