# Supplementary material for: Characteristics of cattle in chronic pens at 17 North American feedlots
Source: Front Vet Sci. 2026 Oct 12;13:1947975. doi: 10.3389/fvets.2026.1947975 (PMC13697008; doi:10.3389/fvets.2026.1947975)
Supplement: Supplementary file 1 [file Table_1.DOCX]

# **Supplementary Material**

**Supplementary Table 1.** Overview of the variables retrieved or created from the database and used in this study, including their descriptions. Lot-level data was retrieved for all lots of cattle placed in the 17 feedlots during the 2014-2020 study period. Individual-level data was retrieved for the 68,645 chronic cattle identified during the study period, and includes variables pertaining to animal characteristics, lifetime treatment history, and health outcomes.

| Variable group | Variable name | Variable description^1^ |
| --- | --- | --- |
| Feedlot Level Data | Feedlot ID | Unique feedlot identifier |
|  | Country | Country of the feedlot of origin |
|  | Feedlot capacity | Average number of head per year for each feedlot |
| Lot Level Data | Population | Number of cattle in the lot. Used to calculate feedlot population for chronicity rates calculation |
|  | Average arrival weight | Average animal arrival weight of the lot (lbs.) |
|  | Females | Number of females |
|  | Males | Number of males |
|  | Low risk | Number of "low" Bovine Respiratory Disease (BRD) risk animals |
|  | High risk | Number of "high" BRD risk animals |
|  | Ship | Number of animals shipped for regular slaughter |
|  | Rail | Number of animals shipped for salvage slaughter |
|  | PM | Number of animals that died at the feedlot |
|  | Euthanized | Number of animals euthanized |
|  | Resp treatment | Number of animals with a respiratory treatment specific diagnosis: Arrival Fever, Diphtheria, No Fever, Undifferentiated Fever |
|  | Lame treatment | Number of animals with a lameness treatment specific diagnosis: Arthritis, Footrot, Foot Lesion/Toe- Tip Necrosis Syndrome, Laminitis, Papillomatous Digital Dermatitis |
|  | MSI treatment | Number of animals with a musculoskeletal (MSI) injury treatment specific diagnosis: musculoskeletal injuries due to facilities, equipment, or other |
|  | ME treatment | Number of animals with a metabolic (ME) treatment specific diagnosis: Atypical Interstitial Pneumonia, Bloat, Overload/Acidosis |
|  | Other treatment | Number of animals with a treatment specific diagnosis other than Resp, Lame, MSI, ME, and Buller. There were 17 different diagnoses fell in this group. The most common were abscess/cellulitis, miscellaneous, nervous disease, congestive heart failure, and eye disease |
|  | Buller treatment | Number of animals with a buller treatment specific diagnosis. A treatment event for these cattle involved moving them from their home pen into a separate specialty pen |
|  | Infectious mortality | Number of animals with an infectious mortality diagnosis: Bronchointerstitial Pneumonia, Bronchopneumonia, Chronic Pneumonia, Chronic Pleuritis and Bloat, Chronic Pneumonia – Mycoplasma, Fibrinous Pneumonia, Infectious Bovine Rhinotracheitis, Lung Abscess, Other Pneumonia, Pneumonia and Arthritis, Viral Interstitial Pneumonia, Chronic Myocarditis, Chronic Pericarditis, Chronic Fibrotic Pleuritis, Diphtheria, Infectious Thrombotic Meningoencephalitis, Myocarditis, Pericarditis, Pleuritis, Septicemia |
|  | ME mortality | Number of animals with a metabolic (ME) mortality diagnosis: Atypical Interstitial Pneumonia, Bloat, Chronic Rumenitis, Enterotoxemia, Frothy Bloat, Laminitis, Liver Abscess, Overload/Acidosis, Posterior Vena Caval Thrombosis |
|  | Lame mortality | Number of animals with a lameness mortality diagnosis Arthritis, Footrot, Foot Lesion/Toe-Tip Necrosis Syndrome, Papillomatous Digital Dermatitis |
|  | Other mortality | Number of animals with a “miscellaneous” mortality diagnosis: cause of death other than Infectious, ME, Lame, MSI, or Buller. There were 48 different mortality diagnoses fell in this group. The most common were no mortality diagnosis (no postmortem performed, unsuitable photos or specimens, no visible lesions, etc.), congestive heart failure, peritonitis, abscess/cellulitis, and embolic pneumonia |
|  | MSI mortality | Number of animals with a musculoskeletal mortality diagnosis, which stem from musculoskeletal injuries due to facilities, equipment, or other |
|  | Buller mortality | Number of animals with a buller mortality diagnosis |
| Individual Level - Demographic Data | Animal ID | Unique animal identifier |
|  | Arrival date | Date of arrival at the feedlot |
|  | Arrival year | Year of arrival at the feedlot; created from arrival date variable |
|  | Arrival season | Season when animal arrived at the feedlot, created from arrival date variable and defined by astronomical season boundaries |
|  | Out date | Date of exit from the feedlot. Includes exit day, week, quarter, month, season, and year |
|  | DOF | Days on Feed (DOF) |
|  | Arrival weight | Arrival weight (lbs) |
|  | BRD risk status | Risk level classification for BRD at feedlot arrival |
|  | Final disposition | Final disposition of each individual animal (Ship, Rail, Mortality) |
|  | Predominant diagnosis group | Treatment diagnosis group (respiratory, lame, metabolic, musculoskeletal, buller, other, or multiple)  with most treatment events received. The conditions included in each treatment diagnosis group are given in the Lot Level Data above. Cattle with equal number of treatments for two or more conditions were classified as "Multiple” |
| Individual Level – Summary of Treatment Data | Treatment total | Total number of treatments received at the feedlot |
|  | First treatment to out interval | Number of days between first treatment and feedlot exit |
| Individual Level –Treatment Data (retrieved for each separate treatment event) | Treatment date | Date at time of treatment. Used to create treatment month and season variables |
|  | DOF at treatment | DOF at time of treatment |
|  | Specific diagnosis | Diagnosis, according to the treatment categories described in the Lot Level Data above |
|  | Diagnosis group | General diagnosis group for the treatment event (respiratory, lame, metabolic, musculoskeletal, buller, or other), as described in the Lot Level Data above |
| Individual Level – Mortality (PM) Data (where applicable) | PM date | Date of PM (postmortem; mortality) event |
|  | Last treatment to PM interval | Number of days between last treatment and mortality event; created from treatment date and PM date variables |
|  | PM DOF | Number of days between feedlot arrival and mortality event |
|  | PM diagnosis group | Standardized mortality group, as listed in Lot Level Data above, assigned to individual specific postmortem diagnosis. All PMs were diagnosed using standardized methods validated by the consulting company to have high degrees of repeatability between onsite and digital PMs and between DVMs |
|  | Euthanasia status | Indicator for euthanized animals |
